# School-based music and extracurricular engagement as pathways to adolescent learning and wellbeing: mediating roles of self-regulation and school climate

**DOI:** 10.3389/fpsyg.2026.1900830

**Published:** 2026-07-31

**Authors:** Wenjun Tian, Tianyi Qiao

**Affiliations:** 1 College of Music, Ewha Womans University, Seoul, Republic of Korea; 2 Faculty of Sports, Belarusian National Technical University, Minsk, Belarus

**Keywords:** academic achievement, adolescents, extracurricular engagement, mediation analysis, music education, PISA 2018, school climate, self-regulation

## Abstract

**Introduction:**

Music and other creative activities are often recommended as affordable ways to support adolescent development, yet large-scale evidence on the psychological correlates linking such engagement to learning and wellbeing remains limited, especially in high-achieving East Asian systems. Using PISA 2018 data from the four Chinese economies of Beijing, Shanghai, Jiangsu and Zhejiang (B-S-J-Z), this study examines whether self-regulation and school climate statistically account for the associations between two engagement indicators and two outcomes. The engagement indicators are school-reported creative and musical provision (e.g., whether the school offers a band, orchestra or choir) and the availability of musical and cultural resources at home (musical instruments; books on art, music or design); both index opportunity and exposure rather than measured participation. Outcomes are academic achievement and subjective wellbeing.

**Methods:**

The analytic sample comprised 12,058 15-year-old students in 361 schools (weighted population ≈ 992,302). A structural equation model with latent mediators and outcomes was estimated via full-information maximum likelihood, complemented by a weighted, plausible-value-pooled regression-based mediation analysis with bias-corrected bootstrap confidence intervals, a school-clustered bootstrap accounting for the nested design, and a gradient-boosting model for triangulation. Given the cross-sectional data, results are reported as associations, not causal effects.

**Results:**

School-reported provision was associated with achievement directly, with no statistical mediation by self-regulation or school climate. Home musical and cultural resources were associated with both outcomes indirectly—through self-regulation and, to a smaller degree, school climate—and for wellbeing the indirect association exceeded the direct one. Self-regulation was the strongest correlate of wellbeing across all methods, though this association is inflated by conceptual overlap with wellbeing and is interpreted with caution.

**Discussion:**

Musical and creative engagement is associated with adolescent outcomes through different statistical routes depending on where the engagement is situated, and self-regulatory capacity and supportive school relationships are central correlates of wellbeing. Findings are specific to B-S-J-Z and are not generalised to China as a whole. Implications for curriculum policy, measurement and future longitudinal research are discussed.

## 1 Introduction

Schools are now expected both to raise academic performance and to protect student wellbeing, and this combined expectation has become a central concern for education systems and for international comparative assessment. The Programme for International Student Assessment (PISA) widened its scope in the 2018 cycle to consider students’ broader competencies and wellbeing alongside cognitive performance, reflecting the view that learning outcomes and psychological health are connected rather than opposed (OECD, 2020a; Govorova et al., 2020). This question is particularly relevant in high performing East Asian systems, where strong measured achievement has often been accompanied by relatively low reported wellbeing, raising the question of whether academic success comes at a psychological cost (Wu et al., 2022).

Participation in music and in other creative and extracurricular activities has received steady attention as a form of engagement that may protect wellbeing and support development. Evidence links musical training to gains in executive function and self-regulatory capacity (Cai et al., 2025; Lu et al., 2025), and engagement with arts and creative activities has been associated with better adolescent mental health and wellbeing (Goopy and MacArthur, 2025; Hugh-Jones et al., 2025). Recent randomised work in China reports that a school-based music programme improved adolescent mental health (Lin et al., 2025), and reviews describe several routes through which music may support wellbeing, including emotional expression and regulation, stronger social bonds and belonging, and the development of creativity (Chen, 2023). At the same time, the strength and the interpretation of these associations remain debated, since fixed effects and quasi experimental analyses show that some simple associations weaken once confounding is taken into account (O'Flaherty et al., 2022).

One limit of the existing literature is that it has examined the outcomes of engagement more often than the processes that might underlie them. Two constructs are repeatedly proposed as candidate explanatory processes. The first is self-regulation, which covers a student’s capacity to set and pursue mastery goals, to persist, and to recover from setbacks. Self-regulated learning is consistently associated with achievement and, according to self-regulated learning theory, may itself be supported by structured, effortful activities such as music (Ryan and Deci, 2020; Shao et al., 2023). The second is school climate, the relational and disciplinary quality of the learning environment, which has been linked through meta-analysis to both achievement and psychological wellbeing (Wang et al., 2020; Erdem and Kaya, 2024). Whether the association between musical and creative engagement and adolescent outcomes is statistically accounted for by self-regulation, by more supportive school climates, or by neither, remains an open question with implications for how schools invest in the arts. Because the present data are cross-sectional, this question is addressed in terms of statistical mediation, that is, whether these constructs account for the observed associations, and not in terms of causal mechanisms.

This study examines that question using the PISA 2018 data for the four Chinese economies of Beijing, Shanghai, Jiangsu and Zhejiang (B-S-J-Z). The context is both substantively important and methodologically useful. It is important because B-S-J-Z recorded the highest mean performance of any participating system in mathematics, reading and science in 2018, which makes the relationship between achievement and wellbeing especially salient there (OECD, 2020b). It is useful because the scale and design of PISA sampling allow population-level inference using validated scales and plausible-value achievement estimates (Jewsbury et al., 2024; Huang, 2024). The study also separates two locations of engagement that earlier work has often combined: school-reported creative and musical provision, captured by the PISA school creative-activities index, and the availability of musical and cultural resources in the home, captured by household musical instruments and arts books. It is important to be precise about what these indicators represent. The school index measures institutional provision, that is, whether a school offers creative activities, not whether a given student participates in them; the home index measures the availability of resources, not active use. Both are therefore proxies for opportunity and exposure rather than direct measures of participation, a distinction that is carried through the interpretation of every result below.

Three research questions guide the study. First, are school-reported creative and musical provision and the availability of musical and cultural resources in the home associated with adolescent academic achievement and subjective wellbeing in the B-S-J-Z context? Second, do self-regulation and school climate statistically account for these associations, and do the indirect associations differ between achievement and wellbeing and between school-based provision and home-based resources? Third, do the conclusions from a confirmatory structural equation model agree with those from a flexible machine-learning model that makes no assumption of linearity? By combining latent-variable mediation modelling, weighted and plausible-value-pooled estimation suited to the survey design, and machine-learning triangulation, the study aims to give a careful and transparent account of how engagement is associated with learning and wellbeing, while treating all relationships as associational given the cross-sectional design.

## 2 Literature review

This section reviews evidence across five areas: musical and creative engagement and learning; extracurricular participation and adolescent outcomes; self-regulation as a developmental mechanism; school climate as a contextual mechanism; and wellbeing in the East Asian achievement context. It ends by stating the gap that this study addresses and the proposed model.

### 2.1 Musical and creative engagement and learning

Interest in the cognitive and academic correlates of music engagement has grown with stronger research syntheses. A recent three level meta-analysis reports that musical training produces small to moderate improvements in children executive functions, with the most reliable effects for inhibitory control and working memory (Cai et al., 2025). Syntheses focused on preschool and early school samples reach similar conclusions, while noting that effects vary with the type and intensity of training (Lu et al., 2025). Because executive function supports the self-regulatory behaviours used in classroom learning, these findings give a reason to expect that music engagement relates to achievement indirectly, through stronger self-regulation, and not only directly.

Evidence from the Chinese context supports this expectation and points to psychological mediators. A study of Chinese students reported that the association between music education and academic and psychological outcomes was carried by self-efficacy and self-esteem (Sun, 2022). Experimental evidence is also accumulating: a cluster randomised controlled trial in a rural Chinese middle school found that a structured, multi week school based music programme improved adolescent mental health relative to a control group (Lin et al., 2025). Research on national music lessons amongst Chinese university students likewise linked musical engagement to subjective wellbeing, self-esteem and identity (Fu and Tu, 2023). Reviews of mechanisms report that music can affect adolescent psychological wellbeing through emotional expression and regulation, stronger social bonds and belonging, and the development of creativity (Chen, 2023).

### 2.2 Extracurricular participation and adolescent outcomes

Beyond music in particular, structured extracurricular participation has long been associated with positive academic and social and emotional outcomes, although the size and meaning of these associations are debated. A fixed effects panel analysis of Australian adolescents found that strong simple relationships between activity participation and positive development weakened substantially once unobserved confounding was addressed, while some benefits of arts participation remained (O'Flaherty et al., 2022). Related work shows that the quality and the motivation of participation matter, since self-determined motivation for extracurricular activities predicts more favourable academic and wellbeing outcomes, in line with self-determination theory (Ryan and Deci, 2020; Verner-Filion et al., 2025). These findings caution against treating provision on its own as sufficient, and they direct attention to the psychological processes that participation may or may not activate.

### 2.3 Self-regulation as an individual-level resource

Self-regulated learning, which includes goal setting, strategy use, persistence and adaptive responses to difficulty, is amongst the most consistent psychological correlates of academic achievement (Shao et al., 2023). Meta-analytic evidence shows that scaffolding regulated learning improves performance, and that a mastery goal orientation in particular supports adaptive outcomes (Shao et al., 2023; Kong et al., 2023). Self-determination theory offers a motivational explanation for why structured and autonomy supportive engagement can develop into stable self-regulatory dispositions that benefit both performance and wellbeing (Ryan and Deci, 2020). Self-regulation is theorised to relate not only to achievement but also to wellbeing, since the capacity to manage effort and emotion supports resilience and positive functioning. This double relevance makes self-regulation a strong candidate mediator linking engagement to both study outcomes.

### 2.4 School climate as a contextual-level resource

School and classroom climate, which covers disciplinary order, teacher support, feedback and adaptive instruction, has been linked through systematic review and meta-analysis to both academic achievement and psychological wellbeing, usually with small to moderate effect sizes (Wang et al., 2020; Erdem and Kaya, 2024). Structural equation analyses confirm climate dimensions as predictors of achievement (Amsalu and Belay, 2024), and PISA based studies show that material and climate resources shape achievement variation across systems (Trinidad, 2020). In East Asian contexts, disciplinary climate has been identified as an especially strong correlate of performance, and a multilevel analysis of PISA 2018 shows that a positive disciplinary climate helps build the academic self-concept that later supports achievement (Ma et al., 2022; Zhao et al., 2023). School climate is also linked to wellbeing, since supportive climates and a sense of belonging are repeatedly associated with better adolescent psychological outcomes (Liu et al., 2024). School climate therefore serves as a second possible mediator, operating at the contextual level alongside the individual level mechanism of self-regulation.

### 2.5 Wellbeing and the east Asian achievement context

The relationship between achievement and wellbeing is neither simple nor uniformly positive. Network and predictive analyses of PISA 2018 wellbeing data identify a set of individual, relational and school factors that together shape student wellbeing (Govorova et al., 2020), and analyses of Chinese adolescents have repeatedly recorded the coexistence of high achievement with relatively modest wellbeing, sometimes finding that achievement relates negatively to wellbeing once family and school factors are modelled (Guo et al., 2022; Wu et al., 2022). Studies that use PISA 2018 data for the Chinese economies have modelled family capital, school climate and achievement as joint determinants of subjective wellbeing using structural equation modelling, which shows both the relevance of these constructs and the value of latent variable approaches in this context (Wu et al., 2022). This literature supports the present expectation that the paths to wellbeing may differ from the paths to achievement.

The B-S-J-Z context also shapes how engagement and wellbeing should be understood. These four economies are amongst the most academically competitive in the world, and adolescents there face intense examination pressure, together with a large private tutoring or shadow-education sector that competes with families’ time and resources. In this environment, participation in music and the arts is often subordinated to examination preparation, and the availability of musical resources at home may partly index families with the economic and cultural capital to protect such activities, while school-provided creative activities may signal better-resourced schools. These features mean that the associations observed here may reflect the particular incentives and constraints of high-stakes East Asian schooling and should not be assumed to hold in systems with different extracurricular cultures.

### 2.6 Theoretical framework and research gap

The conceptual model that guides this study draws together three established theoretical traditions to justify the choice of mediators and the expected directions of association. From self-determination theory, structured and autonomy-supportive activities, including music and the arts, are held to satisfy basic psychological needs for competence, autonomy and relatedness, and thereby to support both motivation and wellbeing (Ryan and Deci, 2020). From self-regulated learning theory, sustained engagement in effortful, goal-directed activity is expected to strengthen students’ capacity to set goals, monitor progress and persist, which in turn supports achievement and adaptive functioning (Shao et al., 2023). From the literature on arts engagement and wellbeing, active involvement in music and creative activity is theorised to promote wellbeing through emotional expression and regulation, social connectedness and belonging, and experiences of mastery and meaning (Goopy and MacArthur, 2025; Caleon, 2019; Hallam and Himonides, 2022; Sheppard and Broughton, 2020). Taken together, these traditions imply that engagement should be associated with the two outcomes not only directly but also indirectly, through the individual resource of self-regulation and the contextual resource of school climate.

On this basis, self-regulation is positioned as an individual-level mediating resource and school climate as a contextual-level mediating resource, both expected to be positively associated with engagement and, in turn, positively associated with achievement and wellbeing. Because home-based resources are proximal to the individual student whereas school-based provision is a property of the institution, the framework anticipates that home-based resources will be more strongly associated with the individual mediator of self-regulation, while school-based provision may relate to achievement through school-level channels that the student-level mediators do not capture. The model is specified symmetrically across the two outcomes so that the data, rather than the specification, reveal where the indirect associations are concentrated.

Three gaps follow from this review. First, although engagement, self-regulation, school climate, achievement and wellbeing have each been studied, they have rarely been combined within a single model that at the same time separates school-based provision from home-based resources and achievement from wellbeing. Second, much earlier PISA-based work has relied on a single method of estimation, which leaves open whether the conclusions hold under a flexible model that makes fewer assumptions. Third, evidence specific to the B-S-J-Z economies is still limited despite their prominence. These gaps require a model that is specified before estimation, links each construct to theory, and states explicit hypotheses before the empirical analysis is introduced.

### 2.7 *A priori* theoretical model and hypotheses

To remove any ambiguity between a theory-driven and a data-driven design, the analytical model was specified *a priori* from the literature before any empirical estimation was conducted. The selection and ordering of variables were not derived from the observed correlation matrix; rather, they follow four complementary theoretical perspectives. Self-determination theory explains why structured music and creative opportunities may support competence, autonomy and relatedness (Ryan and Deci, 2020; Deci and Ryan, 2000). Self-regulated learning theory explains why sustained engagement in effortful creative activity may be associated with goal setting, persistence and adaptive responses to difficulty (Shao et al., 2023; Zimmerman, 2002). School-climate theory explains why supportive teacher relations, feedback, adaptive instruction and disciplinary order may function as contextual resources for achievement and wellbeing (Wang et al., 2020; Erdem and Kaya, 2024; Bronfenbrenner, 1979). Cultural-capital theory explains why musical instruments and art/music/design books in the home may represent unequal access to symbolic and educational resources (Bourdieu, 1986; DiMaggio, 1982). The empirical analysis therefore tests a theoretically specified mediation model rather than searching the PISA database for post-hoc associations (Table 1).

**Table 1:** Theory-to-construct alignment and hypothesis logic.

Theoretical basis	Conceptual role in this study	PISA operationalisation	Expected pathway
Self-determination theory (Ryan and Deci, 2020; Deci and Ryan, 2000)	Music and creative opportunities may support autonomy, competence and relatedness.	School creative/musical provision; home musical-cultural resources.	Engagement/resources are expected to relate positively to achievement and wellbeing.
Self-regulated learning theory (Shao et al., 2023; Zimmerman, 2002)	Self-regulation is the individual mediating resource.	Work mastery, mastery-goal orientation and resilience.	Engagement/resources –> self-regulation –> achievement/wellbeing.
School-climate theory (Wang et al., 2020; Erdem and Kaya, 2024; Bronfenbrenner, 1979)	School climate is the contextual mediating resource.	Disciplinary climate, teacher support, feedback and adaptive instruction.	Engagement/resources –> school climate –> achievement/wellbeing.
Cultural-capital theory (Bourdieu, 1986; DiMaggio, 1982)	Home musical-cultural resources represent family cultural opportunity.	Musical instruments; books on art, music or design.	Home resources are expected to operate more strongly through self-regulation and school climate.
Arts-engagement and wellbeing literature (Goopy and MacArthur, 2025; Caleon, 2019, Sheppard and Broughton, 2020; Eccles and Barber, 1999)	Creative engagement may support expression, belonging, mastery and meaning.	Wellbeing measured by positive affect, meaning in life and sense of belonging.	Indirect associations are expected to be stronger for wellbeing than achievement.

The analytical model that operationalises the theoretical framework is therefore specified before the empirical analysis and its substantive logic can be stated in terms of the named variables. Two engagement indicators, school-reported creative/musical provision and the availability of musical and cultural resources in the home, are specified as antecedents. Two mediators are specified: self-regulation, a latent variable indicated by work mastery, mastery-goal orientation and resilience; and school climate, a latent variable indicated by disciplinary climate, teacher support, perceived feedback and adaptive instruction. Two outcomes are specified: academic achievement, indicated by the mathematics, reading and science plausible-value means; and subjective wellbeing, indicated by positive affect, meaning in life and sense of belonging. Socio-economic status and sex are included as covariates on every endogenous variable.

Conceptually, each engagement indicator may be associated with each outcome along two kinds of route. An indirect route runs from engagement to a mediator to an outcome: for example, greater availability of musical resources at home may be associated with stronger self-regulation, which is in turn associated with higher wellbeing, so that the product of these two associations is an indirect association. A direct route runs from engagement to an outcome net of the mediators and captures any association not accounted for by self-regulation or school climate. The model is estimated symmetrically for both outcomes, so any asymmetry in the results, such as home resources being associated with wellbeing mainly indirectly while school creative/musical provision is associated with achievement mainly directly, is an empirical finding rather than an assumption built into the specification. Throughout, these routes are interpreted as patterns of association consistent with the theoretical framework, not as demonstrated causal pathways, because the data are cross-sectional.

Based on this theory-driven model, four hypotheses were specified before estimation. H1: School-based creative/musical provision and home musical-cultural resources are positively associated with academic achievement and subjective wellbeing. H2: Self-regulation statistically mediates the associations between musical/creative opportunities and adolescent outcomes, with stronger indirect associations expected for wellbeing than for achievement. H3: School climate statistically mediates the associations between musical/creative opportunities and adolescent outcomes, because supportive teacher relations, feedback, adaptive instruction and disciplinary order are contextual resources for learning and wellbeing. H4: The pathway structure differs by engagement location: home musical-cultural resources are expected to operate mainly through self-regulation and school climate, whereas school-level creative/musical provision is expected to show a more direct association with achievement because it may also reflect broader school-level resources. Consistent with the cross-sectional design, the hypotheses are framed as expected associations, not causal effects.

## 3 Methodology

**Figure 1:**
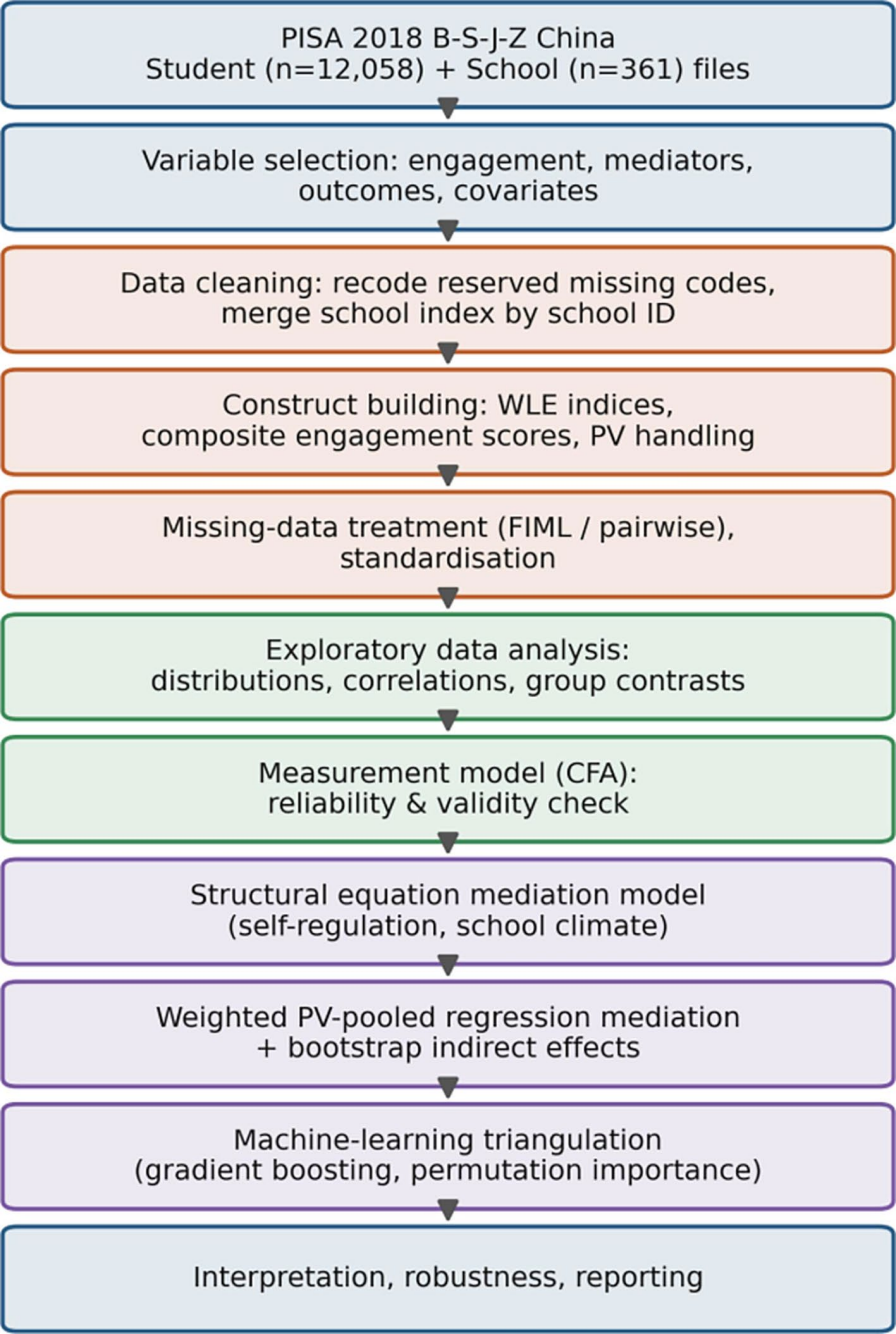
Analytical workflow followed in the study, from data extraction to triangulated reporting.

This study used a cross sectional secondary analysis design applied to the PISA 2018 database for the four participating Chinese economies. The analytic strategy combined a confirmatory latent variable structural equation model, a weighted and plausible value pooled mediation analysis suited to the survey design, and a machine learning model used for triangulation. The overall workflow is summarised in Figure 1. Figure 1 reads from top to bottom and shows the sequence of steps, beginning with the extraction of the student and school files, moving through data cleaning, construct building and missing data treatment, then through the exploratory analysis and the three estimation stages, and ending with triangulation and reporting. Each box represents one stage of the pipeline, and the downward arrows indicate that each stage depended on the output of the stage before it.

### 3.1 Dataset description

The data were drawn from the publicly available PISA 2018 student and school questionnaire databases compiled by the Organisation for Economic Co-operation and Development (OECD, 2020a). PISA assesses 15 year old students using a two stage stratified sampling design in which schools are first sampled with probability proportional to size, and students are then sampled within schools (Jewsbury et al., 2024). Analyses were restricted to the four Chinese economies reported together as B-S-J-Z (Beijing, Shanghai, Jiangsu and Zhejiang). After this restriction, the analytic sample contained 12,058 students in 361 schools. Applying the final student weight gives a weighted population estimate of about 992,302 15 year olds. Females made up 47.8 percent of the unweighted sample.

The identity of the extracted sample was checked against published B-S-J-Z results. Weighted mean performance was 591.4 in mathematics, 555.2 in reading and 590.5 in science, which matches the figures reported in the official PISA 2018 results and confirms that the correct economies had been isolated (OECD, 2020b). Achievement in each domain is represented in the database by 10 plausible values, which are random draws from each student estimated proficiency distribution and which must be analysed together to obtain unbiased point estimates and standard errors (Huang, 2024).

### 3.2 Data preprocessing

Data preparation followed established guidance for secondary analysis of international large scale assessments (Jewsbury et al., 2024). Reserved questionnaire codes for valid skips, not applicable, invalid and no response were recoded to missing before any computation. The school level creative activities index and the indicator of whether a school offers a band, orchestra or choir were merged onto the student records by the unique school identifier, which produced complete linkage for all 12,058 students. Categorical home resource items were converted to binary indicators, and the sex variable was recoded into a female indicator.

Continuous home resource indicators were standardised before they were combined into composite engagement scores, so that each item entered on a common scale. Item level missingness was low for the constructs that were retained, ranging from zero for the school creative/musical provision and achievement variables to about 1.3 percent for positive affect. Two PISA modules were not administered in the Chinese economies. The first is the information and communication technology familiarity module, which contains items on the use of digital devices in music and visual arts lessons. The second is the set of global competence questionnaire items on civic and cultural participation. Both were fully missing and could not be used. In addition, the single life satisfaction item had high missingness and was excluded. These constraints are discussed again as limitations. The structural equation model was estimated by full information maximum likelihood, which uses all available information under a missing at random assumption, while the descriptive statistics and the mediation analysis applied the final student weight.

### 3.3 Measures

Constructs were measured using validated PISA scale indices, which are weighted likelihood estimates, together with selected questionnaire items. The constructs were chosen to fit the proposed model while respecting the items available for the Chinese economies.

The term musical and creative engagement is used in a theory-informed but operationally restricted sense. The PISA 2018 B-S-J-Z data do not contain direct measures of individual participation frequency, duration, intensity or quality in music activities. Therefore, the study does not attempt to define music universally or to measure active musical participation. Instead, it operationalises the construct through observable indicators of opportunity and resource availability: school-reported provision of creative activities, including band, orchestra or choir, and home availability of musical instruments and books on art, music or design. This distinction is central to the study. The school indicator represents institutional provision, while the home indicator represents musical-cultural resources and cultural capital. All interpretations are therefore framed as associations involving opportunities and resources, not as causal effects of music-related opportunity/resource availability.

School-reported creative and musical provision was measured by the PISA school creative-activities index (CREACTIV), a count formed from the school principal’s report of whether the school offers creative extracurricular activities, specifically a band, orchestra or choir, a school play or musical, and art activities (PISA school-questionnaire item set SC053). A binary indicator of whether the school offers a band, orchestra or choir was used as a focused musical marker. Because these items are reported by the school and refer to what the institution makes available, the index measures provision and opportunity, not whether an individual student takes part, and it should not be read as a measure of participation. It is an objective school-level count rather than a psychometric scale, so no internal-consistency coefficient applies.

The availability of musical and cultural resources in the home was measured as the standardised mean of two binary household indicators drawn from the PISA home-possessions items, the presence of musical instruments in the home (ST012Q09) and the presence of books on art, music or design (ST011Q16), with an inter-item correlation of 0.36. This composite indexes a family’s musical and cultural capital and the exposure it affords; like the school index, it captures availability rather than active participation and should be interpreted as a proxy for cultural resources and opportunity. The two-item composite is necessarily narrow, and its cross-cultural validity is limited, because the meaning and prevalence of home musical-cultural resources differ across cultural and economic contexts; this limitation is revisited in the discussion.

Self-regulation was modelled as a latent construct with three indicators, the work mastery scale, the mastery goal orientation scale and the resilience scale, with a composite internal consistency of 0.67. School climate was modelled as a latent construct with four indicators, the disciplinary climate scale, the teacher support scale, the perceived feedback scale and the adaptive instruction scale, with an internal consistency of 0.72. Subjective wellbeing was modelled as a latent construct with three indicators, positive affect, eudaimonia or meaning in life, and sense of belonging, in line with the multidimensional structure of wellbeing in PISA, with an internal consistency of 0.59. Academic achievement was modelled as a latent construct with three indicators, the 10 plausible value means for mathematics, reading and science. The index of economic, social and cultural status and the female indicator were included as covariates throughout.

The psychosocial constructs were operationalised using PISA’s published, internationally scaled indices, which are derived by item-response modelling and scaled to an OECD mean of zero and a standard deviation of one; full construction details are given in the PISA scaling documentation (Huang, 2024). Reliability in the present B-S-J-Z sample, reported in full in Section 5.1, was high for achievement, acceptable for self-regulation and school climate, and modest for wellbeing. Two features warrant caution and are carried through the interpretation. First, the engagement indicators capture provision and availability rather than measured participation, so associations should not be read as effects of participating. Second, the resilience facet of self-regulation and the affective facets of wellbeing share conceptual content, which can inflate the estimated association between the two constructs; this overlap is examined directly in the results and the discussion.

### 3.4 Analytical framework

The analytical framework follows directly from the *a priori* theoretical model presented in Section 2.7. The structural equation model and mediation analysis were used to test the hypothesised pathways, while the machine-learning model was used only as a triangulation tool to examine whether the theory-specified predictors remained important under a flexible non-linear specification.

The analysis proceeded in three stages. In the first stage, a structural equation model with latent mediators and outcomes was estimated by full information maximum likelihood. The measurement part related each latent construct to its observed indicators. The structural part specified the two mediators, self-regulation and school climate, as functions of the two engagement predictors and the covariates, and the two outcomes, achievement and wellbeing, as functions of the mediators, the engagement predictors and the covariates. Model fit was judged using the comparative fit index, the Tucker and Lewis index, the root mean square error of approximation and the goodness of fit index, with the understanding that the chi square statistic is inflated at large sample sizes.

In the second stage, a weighted regression-based mediation analysis provided the primary estimate of indirect effects, because it can apply the survey weight, can pool across all 10 plausible values for the achievement outcome using Rubin rules, and can produce bias corrected bootstrap confidence intervals for the indirect effects (Jewsbury et al., 2024; Huang, 2024; Tibbe and Montoya, 2022). Indirect effects were estimated as the products of the predictor to mediator and the mediator to outcome coefficients, and 95 percent confidence intervals were obtained from 1,000 weighted bootstrap resamples. An effect was treated as reliable when its interval did not include zero.

In the third stage, a gradient-boosting model was fitted for each outcome and evaluated by five-fold cross-validated R-squared, with permutation importance used to rank predictors. This model relaxes the linearity and additivity assumptions of the structural model, and comparing its cross-validated fit with that of ordinary least squares indicates whether non-linear structure is present. The machine-learning stage is included as a complementary, confirmatory check on the theory-driven structural findings, not as an exploratory or purely predictive exercise: its role is to test whether the relative importance of the constructs implied by the structural model is recovered by a method that imposes no functional form.

For transparency, the treatment of the survey design is stated explicitly. Achievement was represented by the 10 plausible values, which were analysed together and pooled using Rubin’s rules (Equation 5), so that both sampling and imputation uncertainty enter the estimates (Jewsbury et al., 2024; Huang, 2024). The final student weight was applied in the descriptive, correlation and regression-based mediation analyses. The structural equation model was estimated by full-information maximum likelihood, which uses all available cases under a missing-at-random assumption; item-level missingness was low, at most about 1.3 percent. Because full-information maximum likelihood in the structural model was fitted without replicate weights, the weighted and nested design was addressed in two complementary ways: the regression-based mediation applied the student weight, and a school-clustered bootstrap (Section 5.4) resampled whole schools so that between-school clustering is reflected in the confidence intervals.

The main estimators are stated below. Bold symbols denote vectors or matrices, and subscripts index variables. Each symbol used in Equations 1–5 is defined in full in the paragraph that follows the equations.


$$z_{\mathrm{ij}}=(x_{\mathrm{ij}}-\mu_{j})/\sigma_{j},E_{i}=(1/p)\sum_{j}z_{\mathrm{ij}}$$ (1)

Equation 1 defines the composite engagement score. The term x with subscript i and j is the raw value of indicator j for student i, the term mu with subscript j is the mean of indicator j across the sample, and the term sigma with subscript j is the standard deviation of indicator j. Their combination z with subscript i and j is the standardised value of that indicator, which places every indicator on a common scale with a mean of zero and a standard deviation of one. The letter p is the number of indicators that make up the construct, and the sum is taken over those p indicators, so the composite engagement score E with subscript i is the average of the standardised indicators for student i.


=Λξ+δ (2)

Equation 2 is the measurement model. The bold x is the vector of observed indicators, the symbol xi is the vector of latent factors that the indicators are intended to measure, the symbol Lambda is the matrix of factor loadings that link each indicator to its latent factor, and the symbol delta is the vector of measurement errors, that is, the part of each indicator not explained by the latent factor.


η=Bη+Γξ+ζ (3)

Equation 3 is the structural model. The symbol eta is the vector of endogenous latent variables, which here are self-regulation, school climate, achievement and wellbeing. The matrix B holds the coefficients for the paths that run from one endogenous variable to another, for example from self-regulation to wellbeing. The symbol xi is the vector of exogenous variables, which here are the engagement predictors and the covariates, and the matrix Gamma holds the coefficients for the paths that run from these exogenous variables to the endogenous variables. The symbol zeta is the vector of structural disturbances, that is, the variance in each endogenous variable not explained by its predictors.


$$\text{Indirect}=a\cdot b;\text{Total}\;c=c^{\prime }+a\cdot b$$ (4)

Equation 4 defines the mediation quantities. The path a is the standardised coefficient from a predictor to a mediator, and the path b is the standardised coefficient from that mediator to an outcome, so their product a times b is the indirect effect that passes through that mediator. The term c prime is the direct effect of the predictor on the outcome after the mediators are included, and the total effect c is the sum of the direct effect and the indirect effect.


a¯=(1/m)∑θk^,T=W¯+(1+1/m)B (5)

Equation 5 states the rules used to combine results across plausible values. The term theta hat with subscript k is the estimate obtained from the k th plausible value, and the pooled point estimate, written as a bar over theta, is the average of these estimates across the m plausible values, where m equals 10 in PISA. The total variance T combines two parts. The within imputation variance, written W bar, is the average of the sampling variances from the separate analyses, and the between imputation variance B measures how much the estimates differ across plausible values. The factor one plus one over m inflates the between part to account for the use of a finite number of plausible values.

### 3.5 Analytical pipeline

The full analytical pipeline is summarised in Algorithm 1. The algorithm lists, in order, every operation carried out in the study, from reading the raw databases to producing the final estimates. Reading the steps in sequence reproduces the workflow shown in Figure 1, but at a finer level of detail, since each numbered step corresponds to a specific computation. Steps 1 to 5 prepare and describe the data, steps 6 to 10 estimate the three models, and steps 11 and 12 combine and report the results.

**Algorithm 1 Integrated estimation and triangulation pipeline.**

1. Input: PISA 2018 student and school questionnaire databases.
2. Restrict the student file to the four Chinese economies (B-S-J-Z), then verify identity against published mean scores.
3. Recode reserved missing codes, then merge the school creative provision index onto students by the school identifier.
4. Construct composite and latent variables, standardise continuous indicators, and form plausible value achievement means.
5. Compute weighted descriptive statistics and internal consistency reliabilities.
6. Estimate the measurement model by confirmatory factor analysis, then assess reliability and fit.
7. Estimate the full structural equation model by full information maximum likelihood, then report standardised paths and fit indices.
8. Estimate weighted regression paths, then pool across the 10 plausible values using Rubin rules.
9. Generate 1,000 weighted bootstrap resamples, then compute indirect effects and 95 percent bias corrected confidence intervals.
10. Fit gradient boosting models for each outcome, then obtain cross validated R squared and permutation importance.
11. Triangulate findings across methods, then interpret them against the hypotheses and report.
12. Output: standardised structural estimates, mediation effects with intervals, and importance rankings.

## 4 Exploratory data analysis

**Figure 2:**
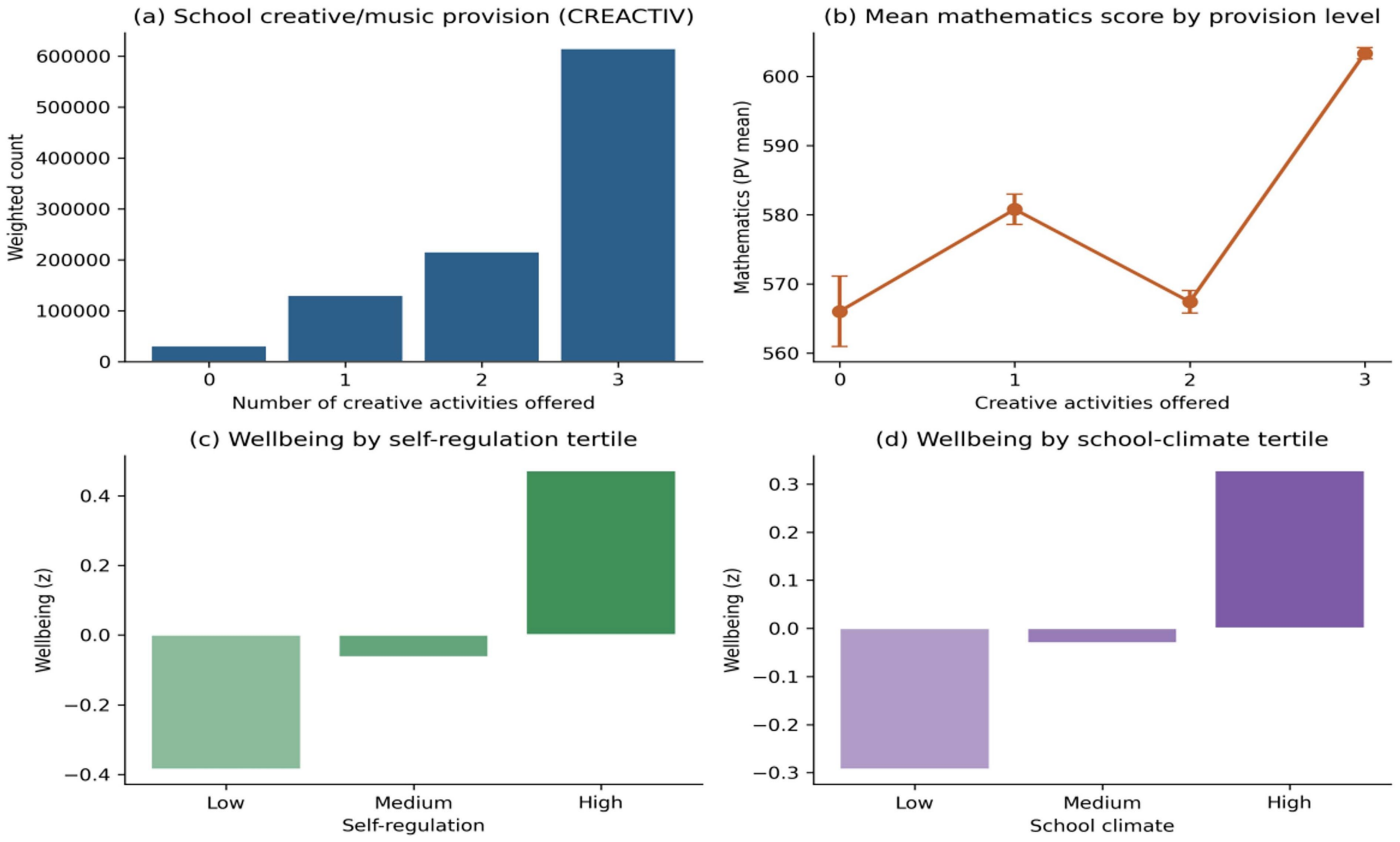
Exploratory views **(a)** distribution of school creative provision; **(b)** mean mathematics score by provision level; **(c)** wellbeing by self-regulation tertile; **(d)** wellbeing by school-climate tertile.

**Figure 3:**
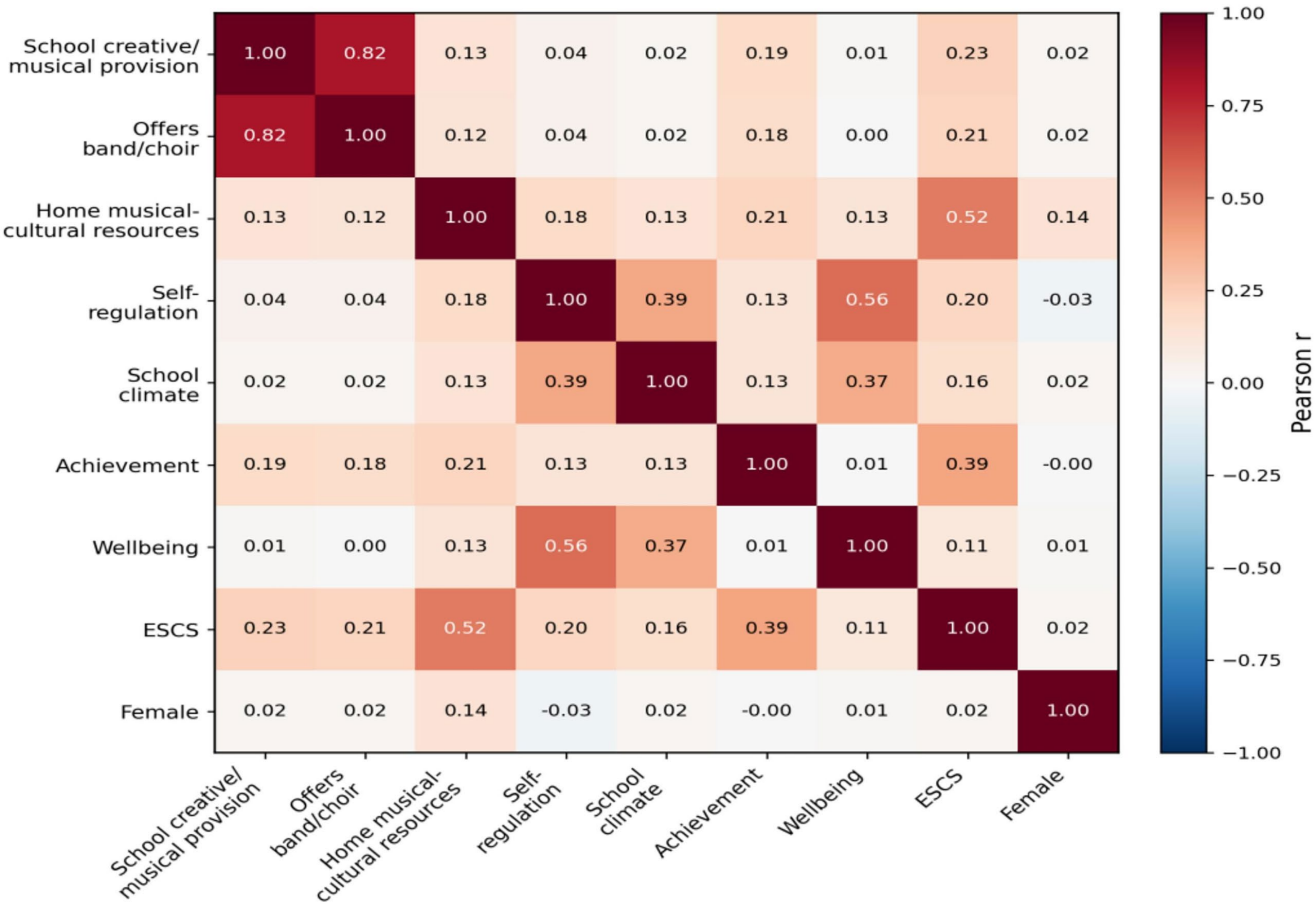
Correlation matrix of the analytic constructs.

Exploratory analysis described the distributions of the engagement measures and the relationships amongst the constructs before the models were estimated. Figure 2 presents four exploratory views, Table 2 reports the weighted descriptive statistics, and Figure 3 together with Table 3 presents the correlation structure. Figure 2 contains four panels. Panel (a) is a weighted histogram of the school creative provision index and shows how many students attend schools offering a given number of creative activities. Panel (b) plots the mean mathematics score against the number of creative activities offered, with vertical bars for the standard errors. Panels (c) and (d) plot mean wellbeing across the low, medium and high tertiles of self-regulation and of school climate, so that the height of each bar shows the average wellbeing of students in that tertile.

**Table 2:** Weighted descriptive statistics for analytic variables (*N* = 12,058 students; 361 schools).

Variable	*N*	Mean	SD	Missing %
Female (proportion)	12,058	0.478	0.5	0.0
ESCS (socio-economic status)	11,990	−0.665	1.071	0.6
School creative/musical provision (CREACTIV)	12,058	2.426	0.833	0.0
School offers band/orchestra/choir	12,058	0.712	0.453	0.0
Home musical-cultural resources	11,989	−0.111	0.811	0.6
Work mastery	11,943	0.276	0.891	1.0
Mastery-goal orientation	11,935	−0.013	0.907	1.0
Resilience	11,982	−0.122	0.938	0.6
Disciplinary climate	11,987	0.793	1.03	0.6
Teacher support	11,988	0.363	0.89	0.6
Perceived feedback	11,968	0.278	1.034	0.7
Adaptive instruction	11,966	0.375	1.037	0.8
Sense of belonging	11,977	−0.19	0.873	0.7
Positive affect	11,896	0.111	0.887	1.3
Meaning in life	11,957	0.078	0.902	0.8
Mathematics	12,058	591.394	73.781	0.0
Reading	12,058	555.236	83.365	0.0
Science	12,058	590.453	77.904	0.0

ESCS = index of economic, social and cultural status. School creative/musical provision and achievement variables had no missing data.

**Table 3:** Correlations amongst principal constructs.

Construct	1	2	3	4	5	6	7	8	9
1. School creative/musical provision	1.0	0.817	0.134	0.043	0.019	0.186	0.01	0.232	0.022
2. School offers band/choir	0.817	1.0	0.125	0.041	0.019	0.179	0.003	0.212	0.02
3. Home musical-cultural resources	0.134	0.125	1.0	0.18	0.134	0.215	0.126	0.522	0.142
4. Self-regulation	0.043	0.041	0.18	1.0	0.388	0.132	0.559	0.205	−0.034
5. School climate	0.019	0.019	0.134	0.388	1.0	0.132	0.371	0.157	0.019
6. Academic achievement	0.186	0.179	0.215	0.132	0.132	1.0	0.007	0.393	−0.003
7. Wellbeing	0.01	0.003	0.126	0.559	0.371	0.007	1.0	0.109	0.01
8. ESCS	0.232	0.212	0.522	0.205	0.157	0.393	0.109	1.0	0.02
9. Female	0.022	0.02	0.142	−0.034	0.019	−0.003	0.01	0.02	1.0

Coefficients with |*r*| ≥ 0.03 are significant at *p* < 0.05 given the sample size. (1) School creative/musical provision; (2) School offers band/choir; (3) Home musical-cultural resources; (4) Self-regulation; (5) School climate; (6) Achievement; (7) Wellbeing; (8) ESCS; (9) Female.

Table 2 reports the weighted mean, standard deviation, sample size and percentage of missing data for every analytic variable. The school creative provision index was high on average, with a weighted mean of 2.43 on its zero to three scale and a standard deviation of 0.83, and about 71 percent of students attended schools that offered a band, orchestra or choir, which indicates that creative provision is common but not universal across B-S-J-Z schools. Figure 2 shows that mean mathematics performance increased steadily with the number of creative activities a school offered, and that both self-regulation and school climate were associated with higher wellbeing in a graded way across tertiles.

Table 3 reports the correlations amongst the principal constructs, and Figure 3 presents the same information as a colour coded matrix in which warmer cells indicate stronger positive correlations. The structure was coherent. School creative provision correlated positively with achievement at 0.19 but only negligibly with wellbeing at 0.01, whereas home musical-cultural resources correlated with achievement at 0.22, with wellbeing at 0.13, with self-regulation at 0.18 and with school climate at 0.13. Self-regulation correlated strongly with wellbeing at 0.56 and moderately with achievement at 0.13, and school climate correlated with wellbeing at 0.37 and with achievement at 0.13. The index of economic, social and cultural status was the strongest single correlate of achievement at 0.39 and was substantially associated with home musical-cultural resources at 0.52, which shows the need to adjust for it. This pattern, in which engagement is linked to the mediators mainly for home based engagement, and the mediators are linked to wellbeing more strongly than to achievement, anticipates the mediation results.

## 5 Results and discussion

### 5.1 Measurement model and construct validity

Before the structural model was estimated, the measurement model was evaluated for reliability and validity following best-practise recommendations for structural equation modelling (Cheung et al., 2024). Table 4 reports, for each latent construct, the range of standardised factor loadings, the composite reliability and the average variance extracted. All loadings were statistically significant. Composite reliability was excellent for achievement at 0.97, acceptable for school climate at 0.73 and for self-regulation at 0.67, and low for wellbeing at 0.56. The average variance extracted was high for achievement at 0.92 but fell below the conventional threshold of 0.50 for self-regulation at 0.41, school climate at 0.42 and, most notably, wellbeing at 0.30. These values indicate that the achievement factor is measured with very high precision; that self-regulation and school climate are acceptable but multifaceted composites formed from heterogeneous weighted-likelihood indices; and that the wellbeing factor, which combines positive affect, meaning and belonging by design, is a broad construct whose indicators share limited common variance. The low reliability and convergent validity of the wellbeing composite is a genuine limitation of the PISA wellbeing indices in this sample; accordingly, estimates that involve wellbeing, and in particular its association with self-regulation, are treated as provisional and interpreted conservatively throughout.

**Table 4:** Measurement model: standardised loadings, composite reliability, and average variance extracted.

Construct	Indicators	Loading range	CR	AVE
Self-regulation	3	0.49–0.77	0.670	0.411
School climate	4	0.41–0.78	0.730	0.416
Academic achievement	3	0.94–0.98	0.971	0.918
Wellbeing	3	0.44–0.63	0.558	0.301

CR = composite reliability; AVE = average variance extracted. All loadings significant at *p* < 0.001.

### 5.2 Structural equation model

**Figure 4:**
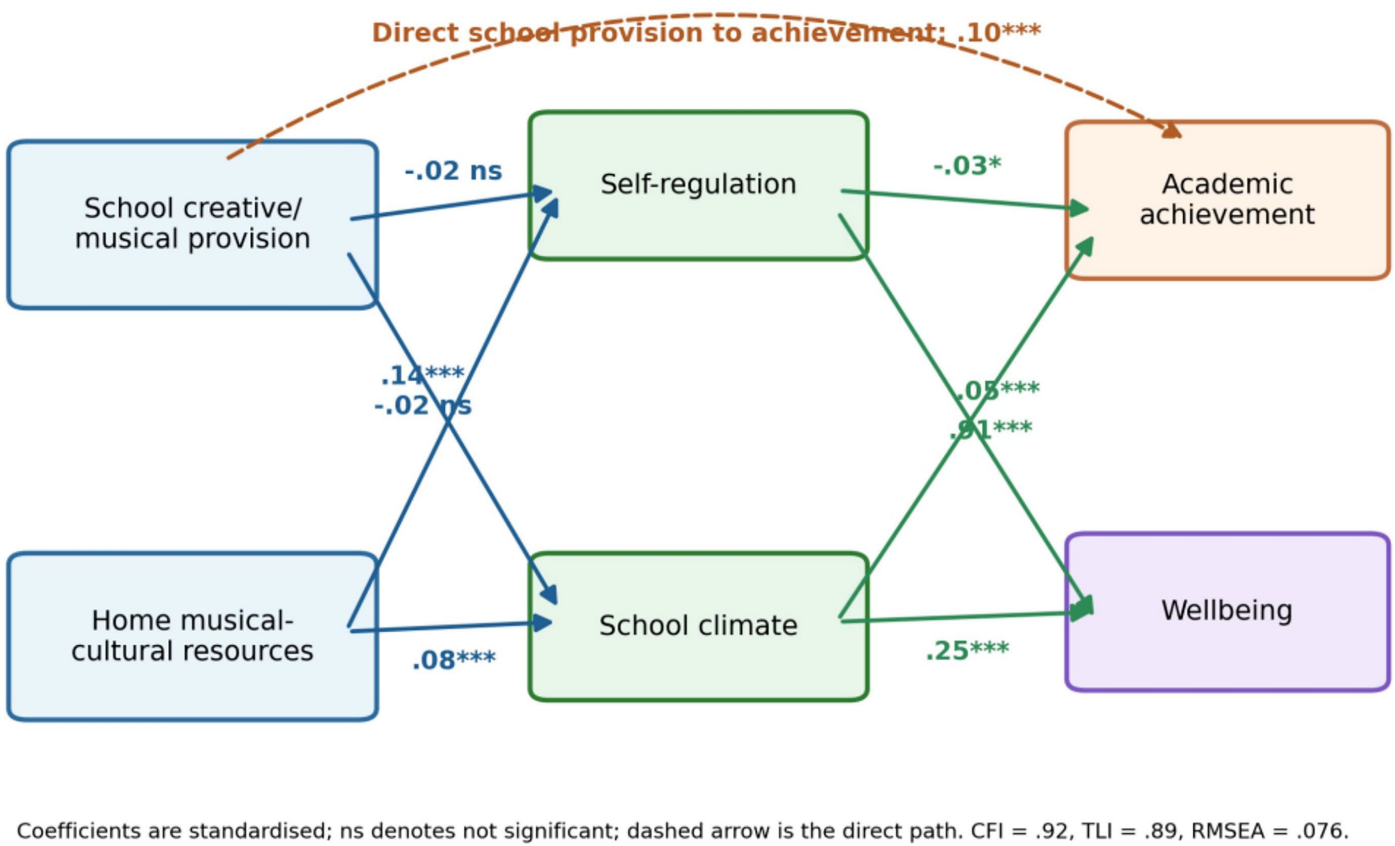
Standardised structural model with latent mediators and outcomes (FIML, *N* = 11,722). Solid arrows are structural paths; the dashed arrow is the direct path from school creative/musical provision to achievement.

The structural equation model converged with acceptable fit given the large sample. The comparative fit index was 0.916, the Tucker and Lewis index was 0.891, the root mean square error of approximation was 0.076 and the goodness of fit index was 0.915, with the chi square statistic of 7,306.84 on 107 degrees of freedom inflated as expected at this sample size. All measurement loadings were substantial and statistically significant, ranging from 0.49 to 0.78 for the mediator indicators, from 0.94 to 0.98 for the achievement indicators, and from 0.44 to 0.63 for the wellbeing indicators, which supports the measurement model. The standardised structural estimates are reported in Table 4 and shown in Figure 4. Table 5 lists each structural path, its standardised coefficient, its *p* value and a label that indicates whether the coefficient was statistically significant. The model accounted for 87 percent of the variance in wellbeing, 17 percent in achievement, 8 percent in self-regulation and 3 percent in school climate, which shows that the predictors explain wellbeing far more fully than they explain the two mediators.

**Table 5:** Standardised structural path coefficients.

Path	Beta (std.)	*p* value	Significant
Self-regulation ← School creative/musical provision	−0.016	0.154	No
Self-regulation ← Home musical-cultural resources	0.138	<0.001	Yes
Self-regulation ← ESCS	0.178	<0.001	Yes
Self-regulation ← Female	−0.099	<0.001	Yes
School climate ← School creative/musical provision	−0.018	0.087	No
School climate ← Home musical-cultural resources	0.083	<0.001	Yes
School climate ← ESCS	0.120	< 0.001	Yes
Achievement ← Self-regulation	−0.025	0.016	Yes
Achievement ← School climate	0.045	<0.001	Yes
Achievement ← School creative/musical provision	0.096	<0.001	Yes
Achievement ← Home musical-cultural resources	0.013	0.192	No
Achievement ← ESCS	0.366	<0.001	Yes
Wellbeing ← Self-regulation	0.914	<0.001	Yes
Wellbeing ← School climate	0.245	<0.001	Yes
Wellbeing ← ESCS	−0.104	<0.001	Yes
Wellbeing ← Female	0.058	<0.001	Yes

Significance is judged at the 0.05 level. Model fit: CFI = 0.916, TLI = 0.891, RMSEA = 0.076, GFI = 0.915.

The model produced three main results. First, school-reported provision was associated with achievement directly, with a standardised coefficient of 0.096 (*p* < 0.001), but it was not associated with either mediator, indicating that this association is not statistically mediated by self-regulation or school climate. This direct association should be interpreted cautiously, because provision is a school-level indicator and may stand for unobserved school-level factors, such as material resources, teacher quality or selective intake, rather than reflecting an effect of provision itself. Second, the availability of home musical-cultural resources was associated with self-regulation (0.138, *p* < 0.001) and with school climate (0.083, *p* < 0.001), establishing the predictor-to-mediator associations required for statistical mediation. Third, self-regulation was the strongest observed correlate of wellbeing (0.914, *p* < 0.001), with school climate also contributing (0.245, *p* < 0.001), while the direct associations from engagement to wellbeing were negligible. This very large coefficient should not be read as evidence that self-regulation is the dominant cause of wellbeing: it is inflated by conceptual overlap between the resilience facet of self-regulation and the affective facets of wellbeing (Section 5.1), so the disattenuated latent association overstates the distinct contribution of self-regulation. Socio-economic status was the strongest predictor of achievement (0.366, *p* < 0.001).

### 5.3 Mediation analysis

**Figure 5:**
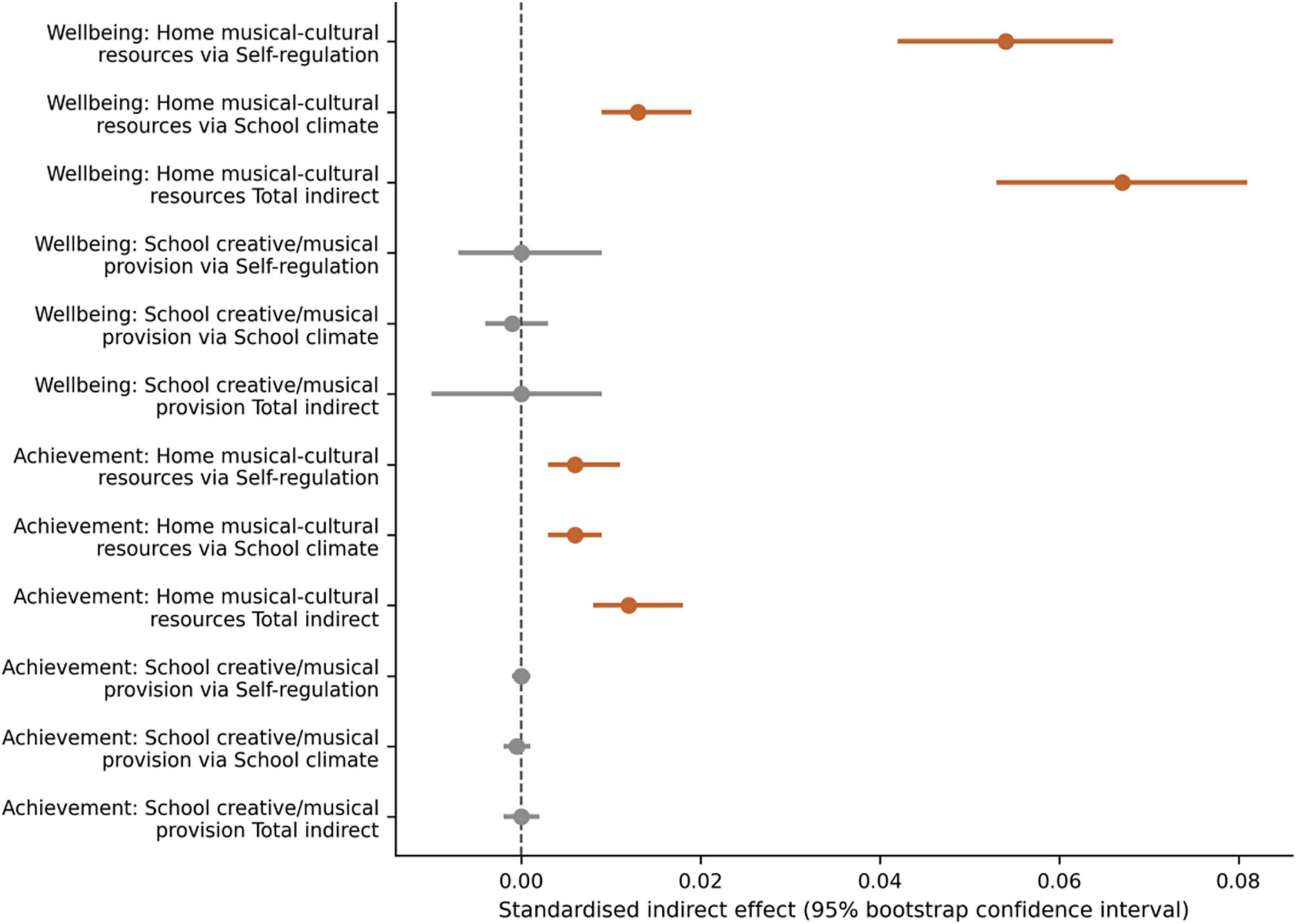
Bootstrapped standardised indirect effects with 95% confidence intervals. Intervals excluding zero (orange) indicate reliable mediation.

The weighted mediation analysis with bootstrap confidence intervals, reported in Table 6 and shown in Figure 5, clarified these paths. Table 6 separates each relationship into its indirect components through self-regulation and school climate, its total indirect effect, and its direct effect, and reports the bootstrap interval for each. Figure 5 presents the same indirect effects as points with horizontal lines for the confidence intervals, where an interval that does not cross the vertical line at zero indicates a reliable effect. For home musical-cultural resources, the total indirect effect on wellbeing was positive and reliable, at 0.067 with a 95 percent interval from 0.053 to 0.081, and it arose mainly through self-regulation, at 0.054 with an interval from 0.042 to 0.066, and to a smaller degree through school climate, at 0.013 with an interval from 0.009 to 0.019. Because the indirect effect was larger than the direct effect of 0.023, the link from home musical-cultural resources to wellbeing was mainly indirect. The indirect effect of home musical-cultural resources on achievement was also reliable but smaller, at 0.012 with an interval from 0.008 to 0.018, divided between self-regulation and school climate.

**Table 6:** Decomposition of effects with 95% bootstrap confidence intervals.

Outcome	Predictor	Effect	Est.	95% CI	Reliable
Wellbeing	Home musical-cultural resources	via Self-regulation	0.054	[0.042, 0.066]	Yes
Wellbeing	Home musical-cultural resources	via School climate	0.013	[0.009, 0.019]	Yes
Wellbeing	Home musical-cultural resources	Total indirect	0.067	[0.053, 0.081]	Yes
Wellbeing	Home musical-cultural resources	Direct	0.023	n/a	n/a
Wellbeing	School creative/musical provision	Total indirect	−0.000	[−0.010, 0.009]	No
Achievement	Home musical-cultural resources	via Self-regulation	0.006	[0.003, 0.011]	Yes
Achievement	Home musical-cultural resources	via School climate	0.006	[0.003, 0.009]	Yes
Achievement	Home musical-cultural resources	Total indirect	0.012	[0.008, 0.018]	Yes
Achievement	School creative/musical provision	Total indirect	−0.000	[−0.002, 0.002]	No
Achievement	School creative/musical provision	Direct path	0.094	n/a	n/a

Estimates are standardised; intervals from 1,000 weighted bootstrap resamples; achievement paths pooled across 10 plausible values.

For school creative provision, neither indirect effect was reliable. The intervals for the total indirect effects on wellbeing, from minus 0.010 to 0.009, and on achievement, from minus 0.002 to 0.002, both included zero. School creative/musical provision instead showed a direct association with achievement, with a coefficient of 0.094. The data therefore show a clear separation. The availability of home musical-cultural resources is associated with the outcomes, and with wellbeing in particular, largely indirectly, that is, via self-regulation and school climate, whereas school-reported provision is associated with achievement directly, through channels that self-regulation and climate perceptions do not capture. These indirect associations are reported as statistical mediation and are not interpreted as causal transmission. This pattern supports the second hypothesis selectively and confirms the differentiated structure expected in the third hypothesis.

### 5.4 Robustness to the nested data structure

Because students are nested within schools, inference that treats students as independent can understate the uncertainty of estimates that involve school-level variation; moreover, the engagement predictor taken from the school questionnaire is constant within schools, and the PISA sample is drawn by first selecting schools and then students. To account for this clustered structure, the bootstrap was repeated by resampling whole schools rather than individual students, a design-based approach that propagates between-school clustering into the confidence intervals and is consistent with guidance for analysing large-scale assessment data with plausible values and weights (Huang, 2024; Tibbe and Montoya, 2022). Table 7 reports the school-clustered indirect associations. The conclusions did not change: the indirect association of home musical-cultural resources with wellbeing remained positive and reliable at 0.068 (95 percent interval from 0.054 to 0.083), as did its indirect association with achievement at 0.013 (95 percent interval from 0.007 to 0.019), while neither indirect association of school creative/musical provision was reliable. The close agreement between the student-level intervals in Table 6 and the school-clustered intervals in Table 7 indicates that clustering does not materially alter the findings. A full two-level structural equation model that explicitly partitions within-school and between-school variance and reports intraclass correlations for each construct would complement this design-based check and is identified as a priority for future work.

**Table 7:** School-clustered bootstrap of total indirect effects (1,000 resamples of schools).

Outcome	Predictor	Indirect	95% CI	Reliable
Wellbeing	Home musical-cultural resources	0.068	[0.054, 0.083]	Yes
Wellbeing	School creative/musical provision	−0.000	[−0.016, 0.015]	No
Achievement	Home musical-cultural resources	0.013	[0.007, 0.019]	Yes
Achievement	School creative/musical provision	−0.000	[−0.004, 0.003]	No

Indirect effects pool self-regulation and school-climate paths; intervals from resampling whole schools to respect the nested design.

### 5.5 Machine learning triangulation

**Figure 6:**
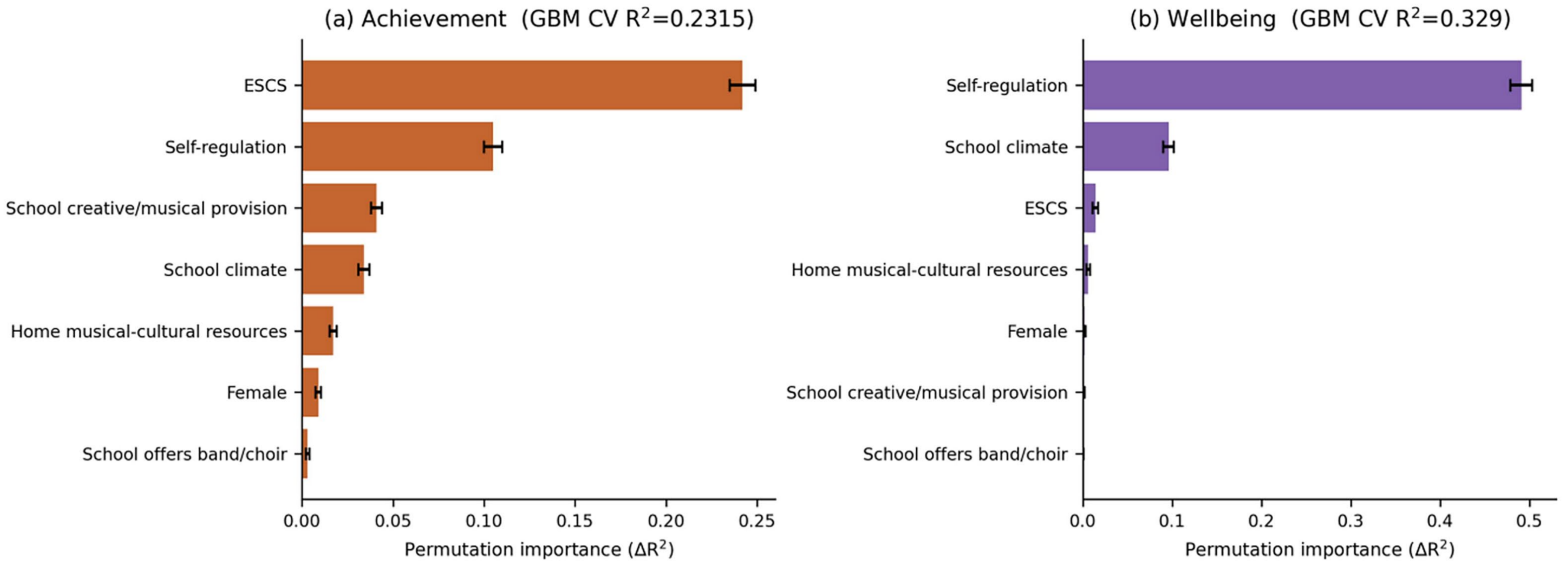
Gradient-boosting permutation importance for achievement **(a)** and wellbeing **(b)**.

The gradient boosting models supported the structural findings. For achievement, the cross validated R squared was 0.232, which exceeded the ordinary least squares value of 0.170 and indicates the presence of non linear structure. Permutation importance ranked the index of economic, social and cultural status first, with a change in R squared of 0.242, followed by self-regulation at 0.105, school creative provision at 0.041 and school climate at 0.034, with home musical-cultural resources contributing a smaller amount at 0.017. For wellbeing, the cross validated R squared was 0.329, close to the linear value of 0.341, which indicates an essentially additive structure. Self-regulation dominated, at 0.491, followed by school climate at 0.096, and the engagement variables contributed little once the mediators were included. Figure 6 displays these rankings in two panels, one for achievement and one for wellbeing, where the length of each bar is the permutation importance of that predictor. The use of a flexible tree-based learner to rank predictors of PISA outcomes is consistent with a growing body of work that applies machine learning to large scale assessment data (Alkan et al., 2025).

This agreement is informative. The prominence of school creative provision for achievement but not for wellbeing in the importance rankings matches the direct provision to achievement path in the structural model and the absence of a provision to wellbeing path. The dominance of self-regulation for wellbeing in both the structural model and the machine learning model increases confidence that self-regulation is the central proximal correlate of wellbeing in this population. The nonlinear structure found for achievement but not for wellbeing suggests that achievement is shaped by interacting resources in a way that a purely linear model understates.

### 5.6 Comparison with prior findings

Table 8 places the present results next to representative prior studies, with one row per study and columns for the context and data, the engagement focus, the mediators and method, and the key finding. The results agree with evidence that music and arts engagement relate to wellbeing and to executive function type capacities (Cai et al., 2025; Hugh-Jones et al., 2025), and with Chinese studies that link music engagement to psychological outcomes through self-related mediators (Sun, 2022). They agree with the wider school climate literature in showing climate to be a reliable, if modest, correlate of both outcomes (Wang et al., 2020; Erdem and Kaya, 2024), and with PISA based Chinese work that models school climate and achievement as joint determinants of wellbeing (Wu et al., 2022). They extend this literature by separating engagement location and by showing, within the highest performing system, that the carrying role of self-regulation and climate is concentrated in the home-resource and wellbeing paths rather than in the school creative/musical provision and achievement path. The weakening of the school creative/musical provision effect on the psychological mediators is also consistent with quasi experimental cautions that provision on its own, without engaged participation, may not activate developmental mechanisms (O'Flaherty et al., 2022).

**Table 8:** Comparison of the present study with representative prior work.

Study	Context and data	Engagement focus	Mediators and method	Key finding
Sun (2022)	China, survey	Music education	Self-efficacy, self-esteem	Mediated psychological and academic outcomes
Cai et al. (2025)	Meta-analysis	Musical training	Executive function	Small to moderate executive-function gains
Wang et al. (2020)	Meta-analysis	Classroom climate	n/a	Climate linked to achievement and wellbeing
Erdem and Kaya (2024)	Meta-analysis	School climate	n/a	Small positive climate to achievement link
Ma et al. (2022)	PISA 2018, B-S-J-Z	Feedback, climate	Self-concept (multilevel SEM)	Climate supports self-concept then achievement
Wu et al. (2022)	PISA 2018, China	Family capital	School climate, achievement (SEM)	Joint determinants of wellbeing
O'Flaherty et al. (2022)	Australia, panel	Extracurricular	Fixed effects	Simple effects attenuate; arts persist
Present study	PISA 2018, B-S-J-Z	Music and creative (school, home)	Self-regulation, school climate (SEM and ML)	Home engagement mediated; provision direct to achievement

## 6 Discussion

Taken together, the three methods give a consistent account, stated here in associational terms because the design is cross-sectional. Musical and creative engagement is associated with adolescent outcomes in B-S-J-Z China, but the pattern depends on where the engagement is situated. The availability of musical and cultural resources in the home is associated with the outcomes largely indirectly, by way of stronger self-regulation and a more positive view of school climate, which are themselves associated with higher wellbeing and, more weakly, higher achievement; this pattern is consistent with the theoretical framework but does not establish a developmental sequence. School-reported provision, by contrast, is associated with achievement directly, without a detectable indirect association through the measured constructs, and this direct association may partly reflect unobserved features of better-resourced schools. The most stable finding is the strong association between self-regulation and wellbeing, recovered by both the confirmatory and the flexible model, although its magnitude is inflated by construct overlap and should be read as showing that self-regulation is an important correlate of wellbeing rather than its principal cause.

The findings should be read primarily as a test of the proposed theoretical model rather than as a data-mining exercise. The strongest support was found for the theory-derived distinction between home resources and school creative/musical provision. Home musical-cultural resources were associated with wellbeing mainly through self-regulation and school climate, consistent with the view that cultural resources become developmentally meaningful when they are connected to students’ motivational and relational resources. In contrast, school creative/musical provision was directly associated with achievement but was not mediated by the measured psychological constructs, suggesting that provision alone may reflect broader school-level opportunity structures rather than individual participation. This distinction strengthens the theoretical contribution of the study: the same broad domain of music and creative opportunity operates differently depending on whether it is located in the home or the school.

These patterns suggest cautious, differentiated implications for practise, offered as hypotheses for confirmatory research rather than as established effects. For in-school creative/musical provision, simply offering creative activities is associated with achievement but shows no association with wellbeing through the measured constructs; if schools wish creative provision to support wellbeing, the design of activities may matter more than their mere availability, for example by structuring them to build goal-setting, persistence and emotion regulation and to foster belonging. For informal, home-based resources, the association with wellbeing operates mainly through self-regulation, which suggests that supporting families’ access to musical and cultural resources, and helping students turn such resources into regular, self-directed practise, may be a complementary route to wellbeing that does not depend on school creative/musical provision alone. Because these findings come from four highly competitive Chinese economies, they should not be generalised to China as a whole or to systems with different extracurricular cultures, and any application to practise should be validated locally.

## 7 Conclusion

This study used the PISA 2018 B-S-J-Z China data to examine whether self-regulation and school climate statistically account for the associations between musical and creative engagement and the two outcomes of academic achievement and subjective wellbeing, combining latent-variable structural equation modelling, weighted plausible-value-pooled mediation with bootstrap inference, a school-clustered robustness check, and machine-learning triangulation. Three conclusions follow, all associational given the cross-sectional design. First, engagement is associated with outcomes through different statistical routes: the availability of home musical-cultural resources is associated with the outcomes substantially through self-regulation and school climate, especially for wellbeing, whereas school-reported provision is associated with achievement directly. Second, self-regulation is the strongest observed correlate of adolescent wellbeing in this sample, a result stable across methods but inflated by construct overlap and therefore interpreted conservatively. Third, the agreement across methods, together with weighting, plausible-value pooling and school-clustered inference suited to the survey design, supports these conclusions. Substantively, the study suggests that whether musical and creative engagement is associated with wellbeing depends less on provision in itself than on whether students develop self-regulatory capacity and are supported by positive school relationships. These conclusions are specific to the B-S-J-Z economies and are not generalised beyond them.

## 8 Limitations and future work

Several limitations frame the findings and the agenda for future research. First, the design is cross-sectional, so every result is an association; the language of mediation is statistical, and longitudinal or experimental designs are needed to establish temporal order and to address unobserved confounding. Second, the engagement indicators capture school-level provision and home-level availability of resources rather than measured participation, so they cannot speak to the effects of actually taking part in music or the arts; measures of individual participation and dosage are needed. Third, the wellbeing composite showed low reliability and convergent validity, and the resilience facet of self-regulation overlaps conceptually with wellbeing, which inflates their association; purpose-built, discriminant-validated scales should be used in future work. Fourth, although a school-clustered bootstrap was used to account for the nested design, a full two-level structural equation model with reported intraclass correlations was not estimated and would be a valuable extension, and the structural model was fitted by full-information maximum likelihood without replicate weights whereas the descriptive and mediation analyses were weighted. Fifth, two PISA modules were not administered in the Chinese economies, which limited the measurement of engagement and wellbeing, and replication in systems that administered them would broaden construct coverage. Finally, and most importantly for interpretation, the findings are specific to Beijing, Shanghai, Jiangsu and Zhejiang, which are amongst the most academically competitive systems in the world; they should not be generalised to China as a whole or to educational contexts with different extracurricular and examination cultures.

## Data Availability

The data analysed in this study are publicly available from the OECD PISA 2018 database (https://www.oecd.org/en/data/datasets/pisa-2018-database.html). The derived variables, analysis code, and figure generation scripts are available from the corresponding author (WT) upon reasonable request.
